# Aspects, Including Pitfalls, of Temporal Sampling of Flying Insects, with Special Reference to Aphids

**DOI:** 10.3390/insects9040153

**Published:** 2018-11-01

**Authors:** Hugh D. Loxdale

**Affiliations:** School of Biosciences, Cardiff University, The Sir Martin Evans Building, Museum Avenue, Cardiff CF10 3AX, Wales, UK; LoxdaleH@cardiff.ac.uk

**Keywords:** adaptation, behaviour, chromosomal–genetic changes, ecological niche, climate and climate change, habituation, hybridization, pathogens–predators–parasitoids, insecticide resistance

## Abstract

Since the advent and widespread use of high-resolution molecular markers in the late 1970s, it is now well established that natural populations of insects are not necessarily homogeneous genetically and show variations at different spatial scales due to a variety of reasons, including hybridization/introgression events. In a similar vein, populations of insects are not necessarily homogenous in time, either over the course of seasons or even within a single season. This of course has profound consequences for surveys examining, for whatever reason/s, the temporal population patterns of insects, especially flying insects as mostly discussed here. In the present article, the topics covered include climate and climate change; changes in ecological niches due to changes in available hosts, i.e., essentially, adaptation events; hybridization influencing behaviour–host shifts; infection by pathogens and parasites/parasitoids; habituation to light, sound and pheromone lures; chromosomal/genetic changes affecting physiology and behaviour; and insecticide resistance. If such phenomena—i.e., aspects and pitfalls—are not considered during spatio-temporal study programmes, which is even more true in the light of the recent discovery of morphologically similar/identical cryptic species, then the conclusions drawn in terms of the efforts to combat pest insects or conserve rare and endangered species may be in error and hence end in failure.

## 1. Introduction

In the present article, I briefly relate how aspects of the temporal sampling of flying insects (Subphylum Hexapoda, Class Insecta) may dictate what is caught, as well as being potentially fraught with pitfalls due to the possibly erroneous assumption that the species populations/subpopulations in question are physiologically–genetically homogeneous. Most of the examples I cite concern aphids (my own speciality), especially those collected in the 12.2 metre-high network of suction traps operated by the Rothamsted Insect Survey since 1964 [1]. However, many of the concerns I discuss may well relate to other flying insects, and indeed other terrestrial as well as aquatic insects and other living organisms, for which I give some examples.

This is not meant to be a comprehensive overview; rather, it is merely a warning that ecological–evolutionary selective pressures working even over relatively short timescales—i.e., years and decades rather than millennia—may influence what we catch in traps and think are the same entity over extended periods, just as spatially-collected insects were thought to be homogenous until the last 40 years or so (see below).

In this short survey of aspects and potential pitfalls, the topics covered include climate and climate change; changes in ecological niches due to changes in available hosts, i.e., essentially, adaptation events; hybridization influencing behaviour–host shifts; infection by pathogens and parasites/parasitoids; habituation to light, sound and pheromone lures; chromosomal/genetic changes affecting physiology and behaviour and hence pre- and post-zygotic effects; and insecticide resistance.

## 2. Background

Until the advent of high-resolution molecular markers in the 1970s onwards, which were initially protein markers (e.g., allozymes) and later DNA markers, especially mitochondrial DNA (mtDNA) and microsatellites [2], insect species populations were assumed to be predominantly genetically homogeneous over large geographic areas [3]. However, during the last quarter of the 20^th^ century, the concept and reality of so-called ‘cryptic’ species became apparent, especially because of observed differences in—for example—host-adapted forms of plant hoppers (Hemiptera: Delphacidae) as a consequence of their distinct mating calls (e.g., [4,5,6]). Thereafter, as populations were explored genetically, further distinct differences were found, including the discovery of hitherto unexpected heterogeneity within and among such populations, so that these could be distinguished as semi- or totally reproductively isolated subpopulations or demes, especially in herbivorous insects. Classic examples include the hawthorn and apple-preferring forms of the apple maggot fly, *Rhagoletis pomonella* (Walsh) (Diptera: Tephritidae) [7,8] and the alfalfa and red clover forms of the pea aphid, *Acyrthosiphon pisum* Harris (Hemiptera: Aphididae) [9,10,11].

Around the same time, also in aphids, the application of allozymes revealed genetic heterogeneity in local UK populations of the blackberry-grain aphid, *Sitobion fragariae* (Walker). This species host alternates between a woody host (on which the autumnally-produced sexual forms—winged males and wingless females or oviparae—mate and lay cold hardy overwintering eggs, e.g., particularly blackberry or bramble, *Rubus fruticosus* agg. L.) and grasses and cereals (Poaceae)—on which asexual (parthenogenetic) propagation occurs throughout the spring and summer months [12,13,14]. It was also found that cryptic obligate asexual forms of the aphid apparently exist sympatrically within such populations and that these did not return to the overwintering woody host, but rather remained on Poaceae all year round, especially cocksfoot grass, *Dactyls glomerata* L. [14]. Later, it was shown using RAPD (random amplified polymorphic DNA) markers [15] and microsatellites and mtDNA, respectively [16], that sympatric host-preferring forms of *Sitobion* aphids existed in the UK. In addition, some level of introgression occurred between those individuals feeding on wild grasses, mainly *D. glomerata* and assumed to be predominantly *S. fragariae sensu lato* and the forms on cultivated wheat, assumed to be the grain aphid, *S. avenae* (F.) *sensu stricto* [17], a predominantly asexual species [18,19,20]. Interestingly, such hybridization was asymmetrical, with males of one host-adapted form preferring to mate with oviparae of the other form compared with vice versa (cf. [16] for further details and Section 2.2.3 below).

Lastly, in field-based studies of *Sitobion* aphids landing on plots of cereals and grasses in a Latin square arrangement, the use of RAPDs revealed that the winged asexual migrant females landing in the spring had distinct host preferences [21]. Furthermore, laboratory studies, also involving RAPDs, identified inter-morph (male vs. female, winged vs. wingless) differences in the asexual lineages (‘clones’ *sensu lato* [22]) of *S. avenae* and the bird cherry-oat aphid, *Rhopalosiphum padi* (L.) kept under conditions of strict clonal hygiene [23].

All these various studies show that, with the widespread use of such molecular markers employed at different spatial scales ranging from geographic to local, to field plot and finally to colony (as in aphids), increasingly fine scale levels of genetic variation have been detected. This in turn emphasizes the fact that populations, even clonal ones, are changing because of mutation, both small and large scale, i.e., within and between chromosomes. Hence, evolution continues on apace as a result of specialist individuals and populations moving into—or establishing—new niches and adapting to such novel ecological scenarios [24,25]. In some cases, especially including aphids with their fast reproduction resulting from parthenogenesis and involving so-called ‘telescoping of generations’ [26], populations undergo fairly rapid mutational changes, maybe over years and decades, rather than millennia and geological time-scales as hitherto assumed [27] (cf. also [28]).

Because of these kinds of changes, including instances of ‘instant speciation’ brought about by chromosome karyotypic changes such as rearrangements consequent upon fission and fusion and translocations (cf. [29,30,31,32,33] for aphids), insect populations are not just changing and adapting in space, but also of course in time. This may especially be true for pest species such as some kinds of aphids (e.g., cereal aphids) whose secondary hosts are transient (i.e., harvested in the agro-ecosystem), whilst at the same time, populations inhabiting these are also nowadays (especially since the 1960s) subject to highly selective pesticide regimes, in addition to a plethora of other natural selecting forces, i.e., climate, predators, parasites and pathogens.

As a direct result of ongoing adaptive ecological forces, including directional selection leading to resistance to pesticides, and other factors such as hymenopterous parasitism, populations of insects may not only be heterogeneous in space, but also in time. This may have a significant consequence in studies (ecological–chemical, ecological, behavioural, and population genetic) involving trapping live insects in which it is taken for granted that the population under investigation is spatially and temporally homogeneous.

### 2.1. Categories of Traps

Aerial trapping can involve a range of devices: light traps (tungsten filament illuminated glass pyramid traps as designed by C.B. Williams (1889–1981), included whilst working at Rothamsted Experimental Station (now Rothamsted Research), Harpenden, Hertfordshire, UK in the 1930s and 40s), and Robinson mercury vapour illuminated traps [34,35,36]; cf. also [37]; suction traps, especially 12.2 metre-high traps, as initially designed by C.G. Johnson (1906–1994) and L.R. Taylor (1924–2007) working at Rothamsted in the late 1940s and early 50s [38]; aerial tow nets [39]; Malaise traps [40]; and, for aphids especially, yellow water traps [41] and in some cases, yellow or blue sticky traps for insects such as thrips, true flies (Diptera), aphids, psyllids, and Coleoptera such as bark beetles [39,42,43,44,45]; CC (plastic cup) and 3D traps for psyllids [46]; Halbert, pers. com.); and pheromone/kairomone lure traps for moths, beetles, fruit flies, etc., e.g., [47]. Terrestrial, ground-dwelling insects, especially including beetles, are usually caught using pitfall traps [48], whilst soil-dwelling invertebrates are often collected using Berlese/Tullgren funnels [49,50]. As well as these static traps and trapping methods, insects may—and of course often are—sampled in both spatial and temporal surveys using nets, i.e., butterfly nets, sweeps nets, both terrestrial and aquatic; white sampling sheets (illuminated with external light sources); beating trays/sheets, sometimes after the forest canopy above has been treated with pyrethroid insecticide to rapidly knockdown the associated insects [51]; pooters; and portable ‘back pack’ suction traps/aspirators (cf. [52] and references therein; [53]).

### 2.2. Categories of Topics

#### 2.2.1. Climate and Climate Change

It is now well established that climate-related seasonal changes of weather patterns affect the timing of insect behaviours, more especially migration events, including the mass migration of insects such as locusts (Orthoptera: Acrididae) and butterflies and moths (order Lepidoptera) [54,55,56,57]. Furthermore, climatic–environmental cues (temperate and light regimes) trigger physiological–genetic changes in insects such as aphids, which undergo profound morphological changes, seemingly involving the epigenetic switching of regulatory genes [58,59]. Such changes are related to the life cycle and life history, and often in heteroecious species involve changes of plant host from overwintering woody hosts to spring and summer herbaceous hosts, as aforementioned (see Section 2; see also [26,60,61]). They also tend to involve different degrees of specialism [62], so that the sexual and pre-sexual winged migrant aphids returning in the autumn to the woody host (e.g., Rosaceae) tend to be much more specialist than the more polyphagous asexual winged forms migrating in the spring to the secondary host/s, although even these forms tend to be specialist within a particular group of related host plants, e.g., Poaceae or Fabaceae [24,25]. In aphids, the switching of hosts and indeed locating of plant hosts generally is related to plant host phenology [63,64] as well as distribution [65], whist the timing of the spring migration is governed by the preceding minimum January–February temperatures [43,44,63,66], and the intensity and duration of the autumn migration back to the woody overwintering host in host alternating (heteroecious) species by both weather-related and nutritional factors [43,63,66,67,68]. Crowding is also a well-known trigger causing the development of winged individuals within colonies on the secondary host [26,58,67,68,69]. With locusts such as *Schistocerca gregaria* (Forskål) (Orthoptera: Acrididae), phase changes are brought about by rainfall affecting laying and hatching success, in turn influencing crowding (and hence hind leg touching) and triggering, via serotonin, a transformation from the solitary to gregarious forms of the insects [70,71]. In cicadas (infraorder Cicadomorpha, superfamily Cicadoidea), the exact environmental triggers for outbreaks of 13 and 17-year species in the USA have yet to be fully elucidated, although assortative mating leading to the allochronic isolation of the periodic ‘species’ along with the selection for prime numbers due to Allee effects appears to be involved in their maintenance (cf. [5,72,73] for further details). All such environmentally-induced and determined changes will cause different morphs of the trapped insect in question at different times of the year, depending on seasonal variation in weather patterns, i.e., early or late spring or summer, etc.

In the case of longer-term weather patterns, now thought to be influenced by global warming, these can have profound effects on both the timing of insects collected in traps as well as which species are actually trapped at a particular location, as climatic conditions move latitudinally north or south. For example, in aphids, those species that are largely or completely asexual in terms of their life cycle (anholocyclic) were found to be advanced in their phenology as a consequence of increasingly warm European weather patterns compared with species that alternate hosts between a woody overwintering host and herbaceous spring and summer hosts (holocyclic) [63]. As for other insects, since the 1970s, several European species, especially including the Speckled Wood butterfly, *Pararge aegeria* (L.) (Lepidoptera: Satyridae) and the Median wasp, *Dolichovespula media* (Retzius) (Hymenoptera: Vespidae), have extensively increased their range, the former in the UK [74,75,76] and the latter having entered the UK in about 1980 and increased its distribution northwards ever since [76]. Other notable (and indeed notorious) examples of recent invasive insects include the Harlequin ladybird beetle, *Harmonia axyridis* (Pallas) (Coleoptera: Coccinelidae) in 2004 [77] and the Asian hornet, *Vespa velutina* Lepeletier (Hymenoptera: Vespidae) in 2016 [78].

The long-term trapping of insects can also demonstrate changes in distribution/and or population density, as with the monitoring of moths using the Rothamsted UK network of tungsten filament light traps. This has shown an overall decrease of species diversity at some sites (e.g., butterflies and moths [79,80,81,82,83]), trends which may reflect changes in the environment due to natural ecological parameters, e.g., weather, or more worryingly, the effects of pesticides and herbicides and environmental degradation due to intensive agricultural practices, e.g., [82,84,85,86,87]. On a related theme, in a seminal paper, the long-term potential consequences of global warming and how such changes in weather patterns might affect the phenology of host plants and hence the emergence and life-cycle of specific insect herbivores feeding on these are considered in depth (cf. [88] and references therein). However, there are caveats, as shown with aphids. Thus, during the great Rose-grain aphid, *Metopolophium dirhodum* (Walker) outbreak of summer 1979, suction-trap surveying of the outbreak as it unfolded showed a northward peak abundance, starting early-on in the south of France and moving northward, apparently crossing the Channel and continuing onward into northern England and Scotland [89]. However, this may actually reflect a change in the phenology of the secondary host, especially cultivated cereals such as wheat, barley and oats, as the season progressed rather than a physical movement (migration) of insects per se [89]; cf. also [90,91,92].

In some other cases, insects that were thought to be extinct or extremely rare, or even species new to the UK, have been discovered as part of the Rothamsted long-term UK national moth light trap survey or in other light traps, e.g., the recent discovery of the micromoth, *Antispila treitschkiella* (Fischer von Röslerstamm) (Lepidoptera, Heliozelidae), the larvae of which feed on Cornelian cherry dogwood, *Cornus mas* L., trapped in summer 2016 as a winged adult in the gardens of the Natural History Museum, London, using a Robinson 125 W MV light trap. The species appears to be expanding its range northwards in Europe, having arrived in the Netherlands in the early 1990s and in Britain in 2016 [93]; cf. also [94].

Lastly, in the past 40 years or so, radar and, more recently, vertical looking radar (VLR) have been used to monitor the aerial movement of flying insects, including nocturnal migrating species, but of course whilst this information has provided novel insight into such movements, because the insects are not physically trapped as such, their identity is surmised from their wingbeat frequency profiles and hence subtle genetic or other changes cannot be recorded, unless the insects are also physically trapped using aerial nets [95,96,97,98]; cf. also [99].

#### 2.2.2. Changes in Ecological Niches Due to Changes in Available Hosts

Particularly over the past several thousand years, non-native animals, plants, fungi and microorganisms, including dangerous diseases, have been transplanted around the world to the UK, notably—but not exclusively—by earlier settlers, including the Romans, Normans, and perhaps also the Vikings, e.g., [100,101,102], a process that is continuing, enhanced by the rapid increase of global transport, e.g., [103]. As European empires expanded in the 16–19th centuries, and naturalists, especially botanists, scoured distant lands for new, exotic species to bring home, the number of such species has steadily grown so that our gardens, and as a consequence of escapees, the European landscape, including Britain, harbour many organisms as a result of these former huge empires. Hence, for example, the UK is now occupied by a diverse range of foreign plants and, to a lesser extent, animals, including even a species of scorpion, the European yellow-tailed scorpion, *Euscorpius flavicaudis* De Geer, which inhabits cracks in the dockyard perimeter wall at Sheerness Docks, Isle of Sheppey, Kent [104]. Because of such introductions, along with invasives that have arrived either naturally or via human agency (in and on vehicles, airplanes, ships etc.), this has naturally offered new opportunities to a range of insect species, particularly including herbivores and pollinators (cf. [105,106,107]. Such new species are captured in insect traps of one kind or another, as aforementioned in the case of moths (Section 2.2.1). With pollinators such as bees, non-native flowers are not necessarily as favoured compared to native flowers [108].

Interestingly, the successful establishment of an invasive or introduced organism is not necessarily certain; sometimes a species will initially be successful in establishing in a new region or ecosystem, but thereafter declines for whatever reason, perhaps due to competition by related species or disease events. For example, the Median wasp, initially very successful in expanding its range into the UK, has seemingly declined in recent years, perhaps due to direct competition for resources with other native *Vespa*/*Vespula* wasp species (H.D.L., pers. obs.; Adam Hart & Mike Edwards, pers. comm.). Japanese Knotweed, *Fallopia japonica* (Houttuyn) has been very successful in colonizing large swathes of the UK since its introduction here in the 19th century, although successful establishment of a biological control agent, the psyllid *Aphalara itadori* (Shinji) to combat it has proved more challenging [109,110,111]. When the Russian Wheat Aphid, *Diuraphis noxia* (Mordvilko) got into the USA in the mid-1980s, its depredation of cereals was extremely destructive: i.e., ≥65% yield loss of small grain cereals in the Great Plains region [90,112,113]. Now, some 30 years later and with the introduction of natural enemies, especially hymenopterous parasitic wasps, to combat it, e.g., [114,115,116], and as these have adapted to it (not always easy as the aphid colony lives in the tight whorls of the plant making parasitisation difficult except by behaviorally-adapted parasitoid species), its numbers and hence the economic damage caused by it have drastically declined in much of its range since the mid-1990s [113]. According to suction trap data from Idaho, USA, the winged forms of the aphid do not travel very far (probably mostly ≤ 20 miles) and predominantly come from nearby fields (“*Relationships between flight activity and field infestations in Idaho support the hypothesis that suction trap collections indicate emigration from expanding local populations rather than long-distance immigration*” [92]), which may have limited the speed of the initial spread of the aphid on its arrival in the States in 1987 [90]. If so, such a low rate of spread may explain why it took another 80 or so years from its discovery on wild Poaceae in the Caucasus region by A.K. Mordvilko (1867–1938) at the turn of the 20^th^ century [117,118] before it arrived in mid-Europe [119,120,121,122,123].

These examples show that a continuing dynamic is in force, with individual species waxing and waning, both spatially and temporally, and that today’s newly invasive or introduced species may be tomorrow’s critically endangered one, or certainly much less common, as factors like climate, predators and parasitoids and pathogens start to influence the ecology and longer-term survival of the species in question. Of course, the ecology of many insect species is still very much a mystery. Thus, for example, the reason why the shade-loving Satyrid species, the Speckled Wood butterfly, *Pararge aegeria* L., should have undergone a range and population explosion over the last 30 years in the UK during the period of climatic temperature rise [124,125], whereas its congener, the Wall Brown butterfly, *Pararge (Lasiommata) megera* (L.), a sun loving species, has concomitantly declined by 86% since 1976 as found by Van Dyck et al. [126] and is now confined to coastal margins in both the UK and Flanders in Belgium, is far from clear but may relate to global warming causing a “developmental trap”. According to these last authors, “*This formerly widespread, bivoltine (or even multivoltine) butterfly has become a conundrum to conservationist biologists. A split-brood field experiment with L. megera indeed suggests issues with life-cycle regulation decisions at the end of summer. In areas where the species went extinct recently, 100% of the individuals developed directly into a third generation without larval diapause, whereas only 42.5% did so in the areas where the species still occurs. Under unfavourable autumn conditions, the attempted third generation will result in high mortality and eventually a lost or ‘suicidal’ third generation in this insect with non-overlapping, discrete generations*.” [126]. They suggest in an associated article [127] “*In effect, these autumn butterflies are a lost generation, leaving no caterpillars that can survive to become butterflies the following spring*.” Such examples demonstrate that it is sometimes difficult in ecology to apply broad generalizations across species, even in the same genus or closely related genera, and that each has to be studied in a species-specific manner.

#### 2.2.3. Hybridization Influencing Behaviour–Host Shifts

According to Mallet [128], around 10% of animals hybridize in nature. Insects, being the most abundant group of animals on the planet, comprising some 75% of all recorded species [129], are no exception in this respect, and many species are known to hybridize, some to the extent of forming new species. An example is the North American Alpine Lycaenid butterfly, a cross between *Lycaeides (Plebejus) melissa* W.H. Edwards and *L. anna* (W.H. Edwards) (formerly *L. idas anna*) which resulted in an isolated hybrid lineage living in the Sierra Nevada mountains of California [130,131,132,133]. As Nice et al. [133] further state, “*When considering the contribution of ecological processes to hybrid speciation, there is the additional possibility of repeated origins of hybrid species leading to multiple isolated lineages of hybrid origin (multiple origins)*.” Many species of stick insects (Order Phasmatodea) have seemingly arisen by hybridization (e.g., [134]; cf. *Chromosomal/genetic changes* section below).

As is well known in biology, hybrids may have hybrid vigour and may sometimes be fertile, but often they have fitness costs and intermediate behaviours, which may be suboptimal in terms of survival, such that they are selected against. For example, in hybridization experiments with *Drosophila pseudoobscura* Frolova and *D. persimilis* Dobzhansky & Epling, Myers et al. [135] showed that “*the cost of hybridization accrues over multiple generations and reinforcement in this system is driven by selection against hybridization above and beyond the cost of hybrid male sterility; we estimate a fitness loss of >95% relative to the parental species across two generations of hybridization*.” Similarly, in sympatric hybridization of Hawaiian *Drosophila, D. heteroneura* Perkins and *D. silvestris* Basden, hybrids of both sexes were fertile but hybridization was not extensive (1.1% at three sites, i.e., 6 of 528 flies of both species surveyed; [136]). As well as hybridization per se, various levels of genetic introgression may occur, leading, as with the *Sitobion* aphids earlier discussed, to asymmetry in the insects resulting from males and females of the different parent species: “….*at least 81% of S. fragariae-like analysed have mitochondrial DNA of the S. fragariae type, suggesting that female S. fragariae mate male S. avenae but that the reciprocal cross is relatively rare*.” [16]

One assumes that such asymmetry may cause asymmetric associations with the original hosts of the parental species, and this in turn is likely to lead, as a result of assortative mating with directional selection against incorrect host attraction and usage, to newly evolved hybrid/introgressed insects going back to the natal parental hosts more frequently than the alternative host/s, depending on the proportion of parental genes they share, something that is observed in fruit flies of the genus *Rhagoletis* infesting Apple and Hawthorn, respectively [7,8,137] cf. also [138]. In *Rhagoletis*, host specificity is governed by behavioural attraction reinforced by host chemical cues [139,140,141]. In the Alpine blue butterfly hybrids, as discussed by Nice et al. [133]: “*The high-altitude lineages tend to have much higher fidelity to their natal larval host plant, as measured by female oviposition preference, than either of the parental species. Two of the lineages (in the Sierra Nevada and White Mts.) exhibit an unusual lack of egg adhesion that causes eggs to fall off of the host plant following oviposition. This trait appears to be adaptive in the alpine habitat where the above-ground biomass of the host plant senesces at the end of the high-altitude growing season* [142]. *Populations in the Sierra Nevada, Warner, and White mountain ranges are also intermediate in terms of the form of the male genitalia* [143], *whereas males from the Siskiyou mountains are not significantly different from L. anna male morphology*.”

In studies of insects involving trapping, any species indulging in hybridization, especially if it is commonplace, are likely to give a false indication of species population abundance, demography and movements, especially when the genetic nature of the sample so collected has not been surveyed using high-resolution molecular markers. If such markers are not applied, cryptic species or indeed hybrids of one sort or another (introgressed in terms of mtDNA, for example) may be present, and their behaviour/flight behaviour may be anomalous: i.e., they may not fly as fast, long, high or seek the normal host/s, probably because their fundamental biochemistry may have been affected, e.g., their flight muscle enzymes. Clearly, this is an aspect to consider when conducting both spatial and temporal studies of insect species, and sometimes, as in parasitoid wasps (Hymenoptera: Braconidae) hitherto unknown genetic entities, possibly cryptic species, are discovered by chance [144,145,146].

#### 2.2.4. Infection by Pathogens and Parasites/Parasitoids

Insects are infected by a range of microbial pathogens, especially including viruses, bacteria, fungi and some species of protozoans (e.g., trypanosomes by tsetse flies and plasmodium by mosquitoes, for which they are of course renowned vectors; [147]), as well as larger parasites such as nematodes [148,149] and nematomorpha worms [150]. These agents can influence the insect’s physiology and behaviour, including reproductive behaviour. For example, certain plant pathogenic viruses, depending on the particular virus and aphid species in question, can induce in their insect vectors “*negative alterations of feeding behaviour (i.e., decreased phloem sap ingestion) and performance that were both conducive for virus fitness by promoting dispersion after a rapid acquisition*”, as well as in other virus-plant-aphid systems “*enhanced feeding behaviour and performances, [which] were favourable to their [the viruses’] acquisition and further dispersal*”, i.e., virus–plant mediated effects on vector transmission efficiency [151]. In similar vein, fungal entomopathogenic species make their aphid hosts (e.g., sugar beet root aphids, *Pemphigus betae* Doane) or Dipterous hosts (i.e., Tipulids) walk up the stems of grasses where they die [152,153], facilitating the spread of the fungal spores by horizontal transmission when these burst forth from the cadaver, or even in some cases, the living insect [152]; cf. also [154]. Lastly, nematomorph worms alter the brain activity of their cricket and grasshopper hosts, inducing these to commit ‘suicide’ by jumping into water, whereupon the adult worms emerge from the unfortunate animal’s anus to seek out a mate and continue the lifecycle [150,155].

It is also known that hymenopterous parasitoid wasp larvae growing within their aphid and other insect hosts manipulate these [156]. For example, 73% of pea aphids, *A. pisum*, left their host plants when parasitized by *Aphidius ervi* Haliday compared with unexposed aphids [157] and doubtless, when infecting winged aphids, which are known to carry them between hosts to found new colonies [158], may well influence the flight behaviour of their insect hosts to some degree by interfering with their essential genetics–chemistry–biochemistry and physiology, e.g., [159]; cf. [160] for *Heliothis* moths and [161] concerning teratocytes.

With the cereal aphid, *S. avenae*, Walton et al. [162] showed using allozyme markers that 18% were infected by braconid parasitoids in the field in the UK (i.e., *Aphidius uzbekistanicus* De Stefani Perez) within a growing season. Similarly, Traugott et al. [163] demonstrated, also in *S. avenae*, but using DNA markers, ~20% endoparasitism by eight primary parasitoid species in 1061 aphids tested along with ~4% DNA for two hyperparasitoid species. The authors relate that “*In 68.2% of the hyperparasitized aphids, identification of the primary parasitoid host was also possible, allowing us to track species-specific parasitoid-hyperparasitoid links. Nine combinations of primary parasitoids within a single host were found, but only 1.6% of all screened aphids were multiparasitized*.” Walton et al. [158] also demonstrated, using *S. avenae* sampled in a 1.5 m high suction trap (used for catching aphids for Barley Yellow Dwarf Virus, BYDV assessment) and tested using allozymes, that a maximum of ~13% aphids parasitized by *Aphidius ervi* and *A. uzbekistanicus* were caught from late May–mid August 1983, with an average of ~5% joint entomopathogenic fungal and parasitoid infection over the same period. These values represent a small, but significant, proportion of the population of aphids under investigation. If it is true that such a proportion of winged aphids are thus infected and perhaps do not complete their aerial migrations between plant hosts, then the total of winged migrants captured over a given period may be underestimated (n.b., using allozyme markers, the assessment of the number of infected aphids is underestimated at any rate, because these markers cannot detect the egg stage [158], although this can be detected using DNA markers due to the greater sensitivity of these; hence, estimates of percentage parasitism are more accurate [163]).

#### 2.2.5. Habituation to Light, Sound and Sex Pheromone Lures

With the continuing growth of the human population globally with its concomitant exploitation of the natural world—mainly terrestrial but to some extent marine also—the increase of light and noise pollution is now known to have direct effects on the life-cycles and life-styles of animal species, including mammals such as Cetaceans, migrating birds, fishes and insects [164,165,166]. With birds, light pollution has caused some species of songbird such as the European robin, *Erithacus rubecula* (L.) to sing more frequently at night [167], whilst road traffic noise is causing them to sing more loudly in order to have their voices heard by rivals when invading and maintaining new territories and attracting mates [168]. As found by Jensen et al., dolphins are also prone to noise pollution from the engines of ships [169], which can and do have negative impacts on their social lives by “*displacing animals from preferred feeding or breeding habitats and by altering their behavioural time budget*”. These authors also state that “*An additional factor … is that the high frequency noise generated by cavitation [‘a phenomenon whereby air bubbles form and collapse on the edge of fast-moving propeller blades’* [170]) *has the potential to impact foraging toothed whales by masking weak echoes from their echolocation signals, which may have a direct bearing on the fitness of the animal.*” [169]. In insects, it has recently been posited that night-flying moths may be adapting to increasing light pollution in suburban and urban environments, especially streetlights, such that some species are actually habituating to this pollution, and also that it may potentially influence their pollination efficiency by affecting their behaviour [171]. Thus, whilst a particular moth species may, for example, be found less commonly over time at a given collection site, this may not reflect an actual decline in local population density: “*It is possible that artificial night lighting could delay or even prevent the onset of nocturnal activity. While this effect is likely to be localised to the immediate vicinity of light sources, it could negatively affect moth fitness (and hence population growth) and nocturnal pollination”* [171]. Electromagnetic radiation from powerlines, phone masts and radar installations and anthropogenic noise pollution [172] may potentially interfere with the movements of insects during mate location, host finding, and migration, as these apparently do with White Storks, *Ciconia ciconia* (L.) [173] and some bat species [174], although in the latter case, this apparently did not affect the abundance of the insects they were foraging on sampled using Pirbright miniature light-suction traps (PMLT) equipped with 8 W UV light bulbs (cf. also [175] for Diptera).

According to a long-term study of insects at 100 nature reserves in Western Europe since the 1980s, whilst annual species population fluctuations were observed, by 2013, overall species numbers were seen to decline by ~75% [176]. This decline is considered to be due to a variety of reasons, principally climate change and direct poisoning due to the widespread and continuing use of pesticides and indirectly due to herbicides reducing flower abundance and hence the pollen and nectar available to insect pollinators and herbivores. The decline of the latter also of course affects those predators (principally, arthropods such as beetles, spiders and birds) and insect parasitoids feeding on them [177]. There is also some evidence for a direct decline of flying insects due to car strikes [178] (cf. also [179] for birds), although the fact that insects are caught in this way has unexpected direct benefits to birds such as house sparrows, *Passer domesticus* L., which have learnt to exploit this resource in recent years [180]. All such changes in the abundance and behaviour of insects are of course likely to affect their density and hence capture and assessment during both short and long-term population surveys.

#### 2.2.6. Chromosomal/Genetic Changes Affecting Physiology, Behaviour and Pre- and Post-Zygotic Effects

It is now well established that small-scale chromosomal changes and especially large-scale changes such as fissions and fusions resulting from translocations may affect insects, both morphologically and behaviourally as a result of divergent selection over time, perhaps enhanced by assortative mating on the natal and novel host/s, as with the host adapted forms of the fruit fly, *R. pomonella* [7]. Such rapid genetic changes can lead to the evolution of host races/subspecies of insects, perhaps with different pheromone preferences—for example, the European corn borer moth, *Ostrinia nubilalis* (Hübner) [181] and the tobacco-feeding sub-specific forms of the peach-potato aphid, *Myzus persicae* (Sulzer) *sensu lato* [182]; cf. also [27]. It can also cause, as with aphids, the production of obligate asexual lineages, which are in effect new species in the sense that they can no longer back-cross with the original facultative sexual parental population [183]. The snapdragon aphid, *Myzus antirrhinii* (Macchiati) and the highly insecticide resistant R_2_ and R_3_ strains of *M. persicae* bearing the autosomal 1 and 3 translocation may be considered as examples of this phenomenon [29,30,184,185]. Delmotte et al. [186], in a study of the genetics of the bird cherry-oat aphid. *R. padi*, consider that mutation of the gene/s concerned with sexual reproduction may also cause novel asexual lineages of the aphid to arise. In the grain aphid, *S. avenae*, an array of asexual, sexual and intermediate forms are now known to occur in the field [187], which complicates the assessment of the true nature of what is being recorded in suction trap catches unless, that is, high resolution molecular markers such as microsatellites are employed to test this possibility [2].

Because of the rapid nature of some insects’ evolution, it is quite possible that samples of one population may change within a season, as well as between seasons. Such a possibility should always be considered. An example of how quickly a new mutation can quickly spread throughout a population relates to the knockdown resistance (i.e., *kdr*, resistant to pyrethroids) genotypes of the grain aphid, *S. avenae*, which were unknown in the field until 2009, but have now spread throughout the UK as a result of direct positive selection using a range of synthetic pyrethroids within the agroecosystem [188,189]; cf. also below).

In the New World screw-worm fly, *Cochliomya hominovoris* (Coquerel) (Diptera: Calliphoridae), a major pest of cattle, especially in the Americas, sterile male approaches using gamma radiation [190] were initially successful in the field, but later attempts using insects mass-reared in culture proved less so [191]. Ultimately, it was found that the mass-reared flies had undergone a mutation in their α-glycerophosphate dehydrogenase (α-GDH; E.C. 1.1.1.8) flight muscle enzyme such that the wild males were out-competing the laboratory-bred and released flies in the field, with the latter showing fitness costs in terms of flight speed and hence finding and impregnating the available females [191,192,193,194]. In other words, the failure of using the mass-rearing, sterile male technique on this occasion was probably due to the fact that the lab-bred males were inbred and thus represented a small sub-population with an apparent dysfunctional flight muscle enzyme system due to inbreeding depression [191,194].

These various aspects of insect change may be pre-zygotic (e.g., mutation of sex-determining genes) or result from the non-disjunction of chromosomes on the metaphase plate during meiosis when two host adapted forms mate, leading to the non-viability of the egg during development or sterility of the offspring, i.e., post-zygotic [140].

With studies using sex pheromones as lures to trap (usually male) insects, or indeed other pheromones such as kairomones [195], the continuing, perhaps over-zealous, use of such chemicals can lead to the habituation of the insects to the lure. This is known to be the case in the highly polyphagous cabbage looper moth, *Trichoplusia ni* (Hübner) (Lepidoptera: Noctuidae) in experiments by Evenden & Haynes using insects reared in the laboratory in the USA and involving a mutation on the gene/s coding for the sex pheromone [196]. According to these authors “…*the sex pheromone consists of a main component, Z7-dodecenyl acetate (Z7-12:Ac), and five minor components* [197]. *Haynes & Hunt* [198] *discovered an abnormal pheromone phenotype in a laboratory colony of T. ni that was the result of a single, recessive autosomal gene mutation. Mutant females release a pheromone that contains a twenty-fold increase of one minor component, Z9-tetradecenyl acetate (Z9-14:Ac) and a thirty-fold decrease of another minor component, Z5-dodecenyl acetate (Z5-12:Ac). Initially, male T. ni that carried the mutant gene responded like normal males, demonstrating a preference for the normal pheromone. However, after 49 generations within a pure mutant colony, males responded equally well to both mutant and normal pheromones* [199].” Thus, there are genetically based differences in both the female sex pheromone and the male response to this [199].

Here then, due to a spontaneous mutation, the nature and attractiveness of males to the female sex pheromone changed over time, leading to the normal pheromone becoming less effective as an attractant and hence less useful as a mating disruptor than hitherto. As Evenden & Haynes [196] conclude, “*Selection imposed by the normal mating disruptant appears to counter the disadvantage of the mutant females, and preserve that genotype in the population. One mechanism for this response to selection could be that normal females become less apparent in disruptant-treated cages to both normal and mutant males, and mutant females may become relatively more apparent. Because mutant males respond equally well to the normal and mutant pheromone, they would be at an advantage to obtain matings from the more apparent mutant females in pheromone-treated cages*.” (cf. [196] for further details). If this scenario were to happen in the field on a large scale, the trapping results obtained using the normal lure might prove erroneous in terms of the effectiveness of attracting winged cabbage looper males and thus controlling, or certainly assessing, the population density of this important pest of many cash crops in North America and Eurasia [200].

#### 2.2.7. Insecticide Resistance

Since the early 1950s, about 500 species of insects and mites (Class Arachnida, Subclass Acari) have become resistant to one or more insecticides globally, e.g., [201]. Thus, the regular application of pesticides can lead to—and indeed has led to—large-scale population changes, including, as found in aphids, the evolution of one or more insecticide resistant mechanisms (sometimes cross resistant within the same individual [202]), leading to the structuring of such populations, perhaps over a wide geographic area, as in the major global aphid pest, *M. persicae* [203,204,205].

Furthermore, many such resistant genotypes of this species, especially the highly resistant ones (i.e., R_2_ and R_3_ genotypes), may have fitness costs in terms of survival and reproduction, due to the pleiotropic influences of the mutational changes on key physiological mechanisms such as the nervous system, in turn leading to changes in behaviour, including the propensity for winged individuals to fly [189,206]. Such highly resistant aphids can also be less responsive to the attacks of predators and hymenopterous parasitoids due to the aphid’s reduced sensitivity to the aphid alarm pheromone, E-β-farnesene [207]. These fitness costs are very likely to, and indeed do, negatively select against certain resistant genotypes, which only persist so long as the chemicals that originally selected for them in terms of individuals bearing suitably adapted resistance mechanisms (e.g., carboxylesterase, MACE (modified acetylcholinesterase) and knockdown resistance against pyrethroids, *kdr*) continue to be used. When they are not, frequencies of the various genotypes may change drastically over time, sometimes rising dramatically due to positive selection, but declining also in light of negative selection, including climatic factors (e.g., winter conditions), predators and hymenopterous parasitoids, and even becoming extinct over both local and larger geographic areas [204]. In contrast, other more ‘stable’ resistant genotypes, usually of lower resistance status (i.e., R_1_), may persist for years within the agroecosystem [189,203,204,208,209,210]. There is also now good evidence that bees (Hymenoptera: Apocrita) are influenced by neonicotinoid pesticides in the environment, both in terms of their foraging success and survival and hence pollination efficiency [87] as well as addiction to such chemicals, as recently found in bumblebees [211].

## 3. Conclusions

The above synthesis is not meant to be a comprehensive review of the various topics covered; rather, its broad aim is to bring to the reader’s attention the possibility of some of the factors mentioned in distorting the true nature of what is captured in temporally based studies of insects, especially flying insects. From what has been said, it is clear that, as with spatial studies, especially if conducted at different spatial scales, it cannot be assumed that the insect species population/s monitored by whatever means are necessarily homogeneous morphologically, or even genetically and as such behaviourally, in terms of their response to host and sex-based odours. The main ’take-home message’ of this article is that insects, with their often huge populations and short lifecycles, are especially likely to show mutations of one sort or another, sometimes a single gene or at other times more large-scale karyotypic changes, producing so-called ‘hopeful monsters’ [212,213], mutant forms which may appear and spread throughout laboratory and, more importantly, natural populations very quickly [27]. It is the existence of these mutated forms, perhaps cryptic, that is another hurdle that we entomologists must be prepared to acknowledge the possible existence of, and by so doing enhance the likelihood of accurate temporal monitoring, for whatever purpose/s this is done, be it fundamental or applied. It is only by recognizing that we may be in error in some of our much beloved concepts or beliefs, often with little empirical evidence one way or another, that we can of course find out the truth of the entomological system, or systems, on which we are currently engaged. Insects, because of their huge variety of species and forms, act as a wonderful forum to enact our research and, to mix metaphors, a canvas on which to paint a new vision of reality as we continue to explore the incredible and often unimagined levels of biodiversity that we ultimately may find, including finer and finer levels of genetic variation [214,215]. This variation may include epigenetic variation, the ecological–evolutionary importance of which is only now being assessed and appreciated in terms of ‘near-Lamarckian’-type processes leading to genetic–physiological–morphological feedbacks: for example, wing development in aphids as a response to colony crowding and/or predator attack, e.g., [59,216,217,218].
